# Relationship Between Clusters of Chronic Conditions and Disability Trajectories

**DOI:** 10.1093/geroni/igab046.3182

**Published:** 2021-12-17

**Authors:** Tara Klinedinst, Lauren Terhorst, Juleen Rodakowski

**Affiliations:** University of Pittsburgh, Pittsburgh, Pennsylvania, United States

## Abstract

Recent evidence shows that more complex clusters of chronic conditions are associated with poorer health outcomes. Less clear is the extent to which these clusters are associated with different types of disability (basic and instrumental activities of daily living (ADL, IADL) and functional mobility (FM)) over time. This was a longitudinal analysis using the National Health and Aging Trends Study (NHATS) (n = 6,179). Using latent class analysis, we determined the optimal clusters of chronic conditions, then assigned each person to a best-fit class. Next, we used mixed-effects models with repeated measures to examine the effects of group (best-fit class), time (years from baseline), and the group by time interaction on each of the outcomes in separate models over 4 years. We identified 5 chronic condition clusters: “multisystem morbidity” (13.9% of the sample), “diabetes” (39.5%), “osteoporosis” (24.9%), “cardio/stroke/cancer” (4.5%), and “minimal disease” (17.3%). Group by time interaction was not significant for any outcome. For ADL outcome, only time was significant (F3,16249 = 224.72, p < .001). For IADL, both group (F4,5403 = 6.62, p < .001) and time (F3,22622 = 3.87, p = .009) were significant. For FM, both group (F4,5920 = 2.96, p = .02) and time were significant (F3,16381 = 213.41, p < .001). We did not find evidence that any cluster experienced greater increases in disability over time, but all clusters containing multiple chronic conditions had risk of IADL and FM disability. Increased screening for IADL and FM disability could identify early disability and prevent decline.

